# A1C as a Diagnostic Criteria for Diabetes in Low- and Middle-Income Settings: Evidence from Peru

**DOI:** 10.1371/journal.pone.0018069

**Published:** 2011-03-25

**Authors:** J. Jaime Miranda, Antonio Bernabe-Ortiz, Sanja Stanojevic, German Malaga, Robert H. Gilman, Liam Smeeth

**Affiliations:** 1 CRONICAS – Center of Excellence in Chronic Diseases, Universidad Peruana Cayetano Heredia, Lima, Peru; 2 Department of Medicine, School of Medicine, Universidad Peruana Cayetano Heredia, Lima, Peru; 3 Faculty of Epidemiology and Population Health, London School of Hygiene and Tropical Medicine, London, United Kingdom; 4 Epidemiology Unit, School of Public Health, Universidad Peruana Cayetano Heredia, Lima, Peru; 5 Department of International Health, Johns Hopkins Bloomberg School of Public Health, Baltimore, Maryland, United States of America; 6 Área de Investigación y Desarrollo, A. B. PRISMA, Lima, Peru; Mayo Clinic College of Medicine, United States of America

## Abstract

**Objectives:**

To determine the prevalence of type 2 diabetes mellitus, in three groups of Peruvian adults, using fasting glucose and glycosylated hemoglobin (A1C).

**Methodology/Principal Findings:**

This study included adults from the PERU MIGRANT Study who had fasted ≥8 h. Fasting glucose ≥126 mg/dL and A1C≥6.5% were used, separately, to define diabetes. Subjects with a current diagnosis of diabetes were excluded. 964 of 988 subjects were included in this analysis. Overall, 0.9% (95%CI 0.3–1.5) and 3.5% (95%CI 2.4–4.7) had diabetes using fasting glucose and A1C criteria, respectively. Compared to those classified as having diabetes using fasting glucose, newly classified subjects with diabetes using A1C (n = 25), were older, poorer, thinner and more likely to come from rural areas. Of these, 40% (10/25) had impaired fasting glucose (IFG).

**Conclusions:**

This study shows that the use of A1C as diagnostic criteria for type 2 diabetes mellitus identifies people of different characteristics than fasting glucose. In the PERU MIGRANT population using A1C to define diabetes tripled the prevalence; the increase was more marked among poorer and rural populations. More than half the newly diagnosed people with diabetes using A1C had normal fasting glucose.

## Introduction

Diabetes is a global problem [1], however there is limited information about this condition in Latin America [2], [3]. Traditionally, for epidemiological studies, diabetes has been defined using fasting plasma glucose ≥126 mg/dL (≥7 mmol/L) [4], [5]. In 2009, the American Diabetes Association suggested that glycosylated hemoglobin (A1C) could be used as a diagnostic tool for diabetes [6]. In the United States, Selvin et al. [7] found individuals with A1C values of 6% or higher were at higher risk of developing diabetes and that A1C was a marker for cardiovascular disease. These results suggest that A1C may be a superior marker to fasting glucose for characterizing long term diabetes risk. However, recently published findings indicate that A1C levels are higher in black than white persons across the full spectrum of glycemia thus potentially limiting the widespread adoption of A1C to screen for glucose intolerance, indicate the risk for complications, measure quality of care, and evaluate disparities in health [8].

In low-and- middle income countries (LMIC), the increased burden of chronic diseases is largely driven by internal migration and urbanization. The dearth of population-based data on hyperglycemia and diabetes [2], as well as on disease progression and mortality limits our ability to intervene appropriately. Furthermore, ethnic differences have been described in A1C levels, [8], [9], [10], [11] which may affect the appropriateness of A1C in LMIC settings.

To our knowledge the impact of using A1C as a diagnostic criterion for diabetes in LMIC has yet to be investigated. Within the Peru MIGRANT study [12], we compared A1C and fasting glucose for the diagnosis of diabetes in rural, rural-to-urban migrants and an urban population. The specific objective was to estimate the prevalence of type 2 diabetes mellitus in adults using fasting glucose and A1C.

## Materials and Methods

### Ethics statement

Ethical approval for this protocol was obtained from ethics committees at Universidad Peruana Cayetano Heredia in Peru and London School of Hygiene and Tropical Medicine in the UK. Written informed consent was obtained from all participants involved in the study.

### Setting and participants

Cross-sectional survey conducted in 2007–2008 of three population-based groups: rural, people born in Ayacucho who had always lived in a rural environment; rural-to-urban migrants, people born in Ayacucho who migrated from rural to urban areas and currently living in Lima; and, urban, people born and currently living in Lima, specifically in the area called “Pampas de San Juan de Miraflores” in a southern district of Lima. Details of the study design have been reported elsewhere [12]. A single-stage random sampling method was used in all groups. In the rural site, the district of San Jose de Secce in Ayacucho, a census was conducted in mid 2007. The sampling frame for the urban group was derived from the local census, conducted in year 2000, which was updated in 2006 to identify all those who referred to have been born in the department of Ayacucho and were currently living in Lima. From these updated censuses, the sampling frame of adults ≥30years-old was 398, 1785, and 4621 individuals for the rural, rural-to-urban migrants and urban groups, respectively [12].

### Study variables

Data were collected through questionnaires (demographics, migration and medical history), a physical examination and blood collection. Fasting glucose, fasting insulin and A1C were measured in plasma, serum and whole blood, respectively. Insulin resistance (HOMA-IR) was calculated using the HOMA calculator [13], excluding those with diabetes.

Plasma glucose was measured using an enzymatic colorimetric method (GOD-PAP, Modular P-E/Roche- Cobas, Germany), serum insulin using electrochemiluminescence (Modular P-E/Roche- Cobas, Germany) and A1C using high-performance liquid chromatography (D10- BIORAD, Germany), which is traceable to the Diabetes Control and Complications Trial reference study as certified by National Glycohemoglobin Standardization Program (NGSP). All samples were analyzed in a single facility. For quality assurance, the quality of assays was checked with regular external standards and internal duplicate assays and monitored by BioRad (www.biorad.com).

The prevalence (95% confidence intervals [CIs]) of diabetes was determined using the American Diabetes Association (ADA) (≥126 mg/dL) [5] cut-offs for fasting glucose and ≥6.5% for A1C [6]. Participants that were aware of their diabetes condition were excluded from the analysis. IFG was defined using ADA's (≥100 and <126 mg/dL) and WHO's (≥110 and <126 mg/dL) cut-offs for fasting glucose.

### Statistical analysis

The κ statistic was calculated to measure agreement between the two definitions [14]. Comparison of proportions and medians between groups were evaluated through Fisher's exact test and Kruskal-Wallis tests. Data were analyzed using Stata 11 (Stata Corporation LP, College Station, TX).

## Results

A total of 988/989 participants, aged 30–92 years, enrolled in this study had complete information for both fasting glucose and A1C. Twenty-four subjects who were aware of their diabetes condition were excluded from the analysis. Using fasting glucose 9/964 (0.9%; 95%CI 0.3–1.5) were classified as having diabetes, whereas 34/964 (3.5%; 95%CI 2.4–4.7) had diabetes using A1C. Of those with fasting glucose ≥126 mg/dL, none had A1C<6.5%. Fair agreement existed between these diagnostic criteria (κ 0.41; 95%CI 0.23–0.59).

The profile of those newly classified diabetes cases, where A1C was ≥6.5% but fasting glucose was <126 mg/dL (n = 25), were older, more socioeconomically deprived and had higher blood pressure levels (Table 1). The newly diagnosed group was not different from the non-diabetes group in terms of hemoglobin levels, anemia, BMI, smoking, physical activity, cholesterol and insulin levels. 40% corresponded to ADA's IFG cases or 28% using WHO's IFG definition (Table 1).

**Table 1:** Characteristics of the PERU MIGRANT population according to A1C and fasting glucose levels.

		A1C<6.5%*	A1C≥6.5%		
	All	Glucose<126**	Glucose<126**	Glucose≥126**	p-value
	n = 964	n = 930	n = 25	n = 9	
**Demographic and socioeconomic**					
Age, years (mean, SD)¥	47.9 (12.1)	47.5 (11.8)	58.4 (15.7)	55.7 (11.1)	<0.001
Men (%, 95%CI)†	47.2 (44–50.4)	47.4 (44.2–50.6)	40 (19.4–60.6)	44.4 (3.9–85)	0.78
Socioeconomically deprived (%, 95%CI)^1^^,^ ¥	30.7 (27.8–33.6)	29.6 (26.6–32.5)	68 (48.4–87.7)	44.4 (3.9–85)	<0.005
**Cardiovascular risk factors**					
Hemoglobin, g/dL (mean, SD)¥	14.2 (1.6)	14.2 (1.7)	14.7 (1.5)	14.7 (1.7)	0.16
Anemia (%, 95%CI)^2^^,^ †	7.9 (6.2–9.6)	8.1 (6.3–9.8)	4.0 (0.0–12.3)	–	0.86
BMI, Kg/m^2^ (mean, SD)¥	26.5 (4.6)	26.4 (4.5)	25.7 (5.9)	30.9 (6.3)	0.02
Current smoking (%, 95%CI)^3^^,^ †	11 (9–13)	11.1 (9.1–13.1)	8.0 (0.0–19.4)	11.1 (0.0–36.7)	0.99
Low physical activity (%, 95%CI)^4^^,^ †	26.0 (23.3–28.8)	26.1 (23.3–28.9)	20.8 (3.3–38.4)	33.3 (0.0–71.8)	0.76
Systolic blood pressure, mmHg (mean, SD)¥	121.6 (18.6)	121.1 (18.1)	133.1 (25.7)	141.7 (28.7)	<0.01
Diastolic blood pressure, mmHg (mean, SD)¥	72.8 (9.9)	72.6 (9.7)	79.7 (12.7)	83.2 (12.4)	<0.001
Total cholesterol, mg/dL (mean, SD)¥	184.1 (40.8)	183.5 (40.0)	185.0 (56.5)	238.7 (41.7)	<0.01
HDL-cholesterol, mg/dL (mean, SD)¥	44.1 (11.6)	44.1 (11.5)	43.8 (11.8)	45.7 (20.7)	0.79
LDL-cholesterol, mg/dL (mean, SD)¥	110.2 (34.4)	110.0 (33.9)	108.1 (45.2)	142.8 (38.1)	0.03
Tryglicerides, mg/dL (mean, SD)¥	152.4 (93.3)	150.5 (91.4)	165.9 (90.4)	304.9 (161.8)	<0.01
**Metabolic markers**					
Fasting glucose, mg/dL (median, IQR)¥	85 (79–91)	85 (79–90)	96 (84–112)	148 (134–256)	<0.001
A1C, % (median, IQR)¥	5.6 (5.4–5.9)	5.6 (5.3–5.8)	6.7 (6.5–7.0)	8.2 (7.3–12.7)	<0.001
Insulin, µIU/mL (median, IQR)¥	6.0 (3.3–9.9)	5.9 (3.4–9.8)	5.2 (1.9–12.6)	9.1 (6.9–17.7)	0.07
HOMA-IR (median, IQR)^5^^,^ ¥	0.8 (0.4–1.3)	0.8 (0.4–1.3)	0.7 (0.3–1.7)	1.6 (1.1–2.7)	0.01
**Impaired fasting glucose**					
IFG ADA – %, (95%CI)^6^^,^ †	7.4 (5.8–9.2)	6.6 (5.1–8.4)	40 (21.1–61.3)	–	<0.001
IFG WHO – %, (95%CI)†	1.5 (0.8–2.4)	0.8 (0.3–1.6)	28 (12.1–49.4)	–	<0.001

**Notes:**

*No cases matched the criteria for the group A1C<6.5% and Glucose≥126 mg/dL.

**Unit of fasting blood glucose in mg/dL.

1 At least 2 or more socioeconomic deprivations from four areas: educational level (none or incomplete primary education), household income (less than USD $150 dollars per month) and asset's possession (lowest tertile of possessions weighted asset index).

2 Anemia was defined as having hemoglobin <12 among females or <13 among males.

3 Current smoking was defined as having smoked within the last six months and a lifetime total of more than 100 cigarettes.

4 Low physical activity was defined as those participants with <600 MET minutes per week. Information on physical activity was available for 956/964 subjects.

5 Information for HOMA-IR was available on 953/964 subjects.

6 ADA's IFG, fasting glucose≥100 and <126 mg/dL. WHO's IFG, fasting glucose≥110 and <126 mg/dL.

†p-values were obtained comparing between the three groups using Fisher's exact test.

¥p-values were obtained comparing between the three groups using Kruskal-Wallis test.

The profile of those with raised A1C but low fasting glucose using standard IFG classifications is shown in Table 2. Participants with raised A1C and IFG were more likely of not being socioeconomically deprived and to have higher BMI, higher blood pressure, higher insulin and higher HOMA-IR.

**Table 2:** Characteristics of participants newly classified as diabetes cases based on ADA and WHO's cut-offs for IFG.

	ADA's IFG cut-off			WHO's IFG cut-off		
	≥100 and <126 mg/dL			≥110 and <126 mg/dL		
	No IFG	IFG	p-value	No IFG	IFG	p-value
	n = 15/25	n = 10/25		n = 18/25	n = 7/25	
**Demographic and socioeconomic**						
Age, years (mean, SD)¥	58.7 (14.0)	58 (18.6)	0.87	57.7 (14.5)	60.3 (19.4)	0.65
Men (%, 95%IC)†	33.3 (11.8–61.6)	50 (18.7–81.3)	0.44	27.8 (9.6–53.4)	71.4 (29–96.3)	0.08
Socioeconomically deprived (%, 95%CI)^1^^,^ †	86.7 (59.5–98.3)	40 (12.2–73.8)	0.03	83.3 (58.6–96.4)	28.6 (3.7–71)	0.02
**Cardiovascular risk factors**						
Hemoglobin, g/dL (mean, SD)¥	14.7 (1.6)	14.8 (1.3)	0.96	14.8 (1.6)	14.6 (1.1)	0.81
Anemia (%, 95%CI)^2^^,^ †	6.7 (0.0–21.0)	0.0 (0.0–30.8)	0.60	5.6 (0.0–17.3)	0.0 (0.0–41.0)	0.72
BMI, Kg/m^2^ (mean, SD)¥	24.2 (5.1)	27.9 (6.6)	0.11	24.1 (4.9)	29.6 (6.8)	0.04
Current smoking (%, 95%CI)^3^^,^ †	6.7 (0.2–31.9)	10.0 (2.5–44.5)	0.99	5.6 (0.1–27.3)	14.3 (0.4–57.9)	0.49
Low physical activity (%, 95%CI)^4^^,^ †	26.7 (7.8–55.1)	11.1 (2.8–48.3)	0.62	22.2 (6.4–47.6)	16.7 (0.4–64.1)	0.99
Systolic blood pressure, mmHg (mean, SD)¥	122.8 (15.5)	148.5 (30.7)	0.04	123.5 (14.3)	157.7 (32.8)	0.02
Diastolic blood pressure, mmHg (mean, SD)¥	74.8 (9.9)	86.9 (13.3)	0.04	75.3 (9.4)	90.9 (13.7)	0.02
Total cholesterol, mg/dL (mean, SD)¥	171.6 (49.4)	205.2 (63.0)	0.16	178.6 (61.1)	201.6 (42.2)	0.24
HDL-cholesterol, mg/dL (mean, SD)¥	47.1 (12.5)	38.7 (9.1)	0.10	44.6 (13.5)	41.6 (5.6)	0.49
LDL-cholesterol, mg/dL (mean, SD)¥	96.4 (33.8)	125.6 (55.8)	0.13	103.9 (50.2)	118.8 (29.3)	0.15
Tryglicerides, mg/dL (mean, SD)¥	140.3 (79.6)	204.4 (96.0)	0.05	150.4 (76.0)	205.9 (117.3)	0.25
**Metabolic markers**						
Fasting glucose, mg/dL (median, IQR)¥	85 (80–93)	116 (109–119)	<0.001	85.5 (82–97)	118 (116–120)	<0.001
A1C, % (median, IQR)¥	6.6 (6.5–7.1)	6.8 (6.6–7)	0.30	6.7 (6.5–7.1)	6.8 (6.6–6.9)	0.60
Insulin, µIU/mL (median, IQR)¥	2.0 (1.5–6.4)	16.3 (5.3–21.5)	0.002	3.5 (1.7–6.4)	16.4 (8.6–21.9)	0.003
HOMA-IR (median, IQR)^5^^,^ ¥	0.3 (0.2–0.9)	2.2 (0.7–2.9)	0.001	0.5 (0.2–0.9)	2.2 (1.2–3.0)	0.003

**Notes:**

1 At least 2 or more socioeconomic deprivations from four areas: educational level (none or incomplete primary education), household income (less than USD $150 dollars per month) and asset's possession (lowest tertile of possessions weighted asset index).

2 Anemia was defined as having hemoglobin <12 among females or <13 among males.

3 Current smoking was defined as having smoked within the last six months and a lifetime total of more than 100 cigarettes.

4 Low physical activity was defined as those participants with <600 MET minutes per week. Information on physical activity was available for 956/964 subjects.

5 Information for HOMA-IR was available on 953/964 subjects.

†p-values were obtained comparing between the three groups using Fisher's exact test.

¥p-values were obtained comparing between the three groups using Kruskal-Wallis test.

The distributions of new classification of diabetes by migration status are shown in Table 3. There were more newly diagnosed diabetes cases, as defined by A1C, in the rural group (6.5%) compared to the migrant and urban groups (1.2% and 2.7%, respectively).

**Table 3:** Characteristics of population according to A1C and fasting glucose levels by migration status.

	A1C<6.5%	A1C≥6.5%		p-value
	Glucose<126*	Glucose<126*	Glucose≥126*	
**Population distribution by study group** (%, 95%CI)†	**n = 930**	**n = 25**	**n = 9**	
Rural (n = 200)	93 (88.5–96.1)	6.5 (3.5–10.9)	0.5 (0.0–2.8)	0.002
Migrant (n = 575)	97.9 (94.6–98.9)	1.2 (0.5–2.5)	0.9 (0.3–2.0)	
Urban (n = 189)	95.8 (91.8–98.1)	2.7 (0.9–6.1)	1.6 (0.3–4.6)	
**Migration patterns (migrant population only)**	**n = 571** **	**n = 7**	**n = 5**	
**Age at migration** (median, IQR)¥	14 (10–17)	17 (11–24)	18 (17–25)	0.02
**Years lived in urban area** (median, IQR)¥	31 (25–39)	35 (25–37)	38 (31–38)	0.87
**Lifetime exposure to urban area** (%, 95%CI)¥	69.6 (59.1–77.8)	71.6 (51.4–78.7)	60.3 (55.4–67.9)	0.31

**Notes:**

*Unit of fasting blood glucose in mg/dL.

**n = 571 for age at migration and n = 546 for other migration classifications.

†p-values were obtained comparing between the three groups using Fisher's exact test.

¥p-values were obtained comparing between the three groups using Kruskal-Wallis test.

## Discussion

When applied to a sample of Peruvian migrant and non-migrant population, the new recommendation by the International Expert Committee [6] to use A1C to diagnose diabetes would result in a tripling of the prevalence of diabetes. Our findings suggest that forty percent of people who would be newly labeled as having diabetes are likely to have normal fasting glucose. The increased prevalence of diabetes will be more marked among lower socioeconomic groups and among rural populations, and no evidence of differences in levels of smoking and anemia we observed. Our study also identified that those that qualify as diabetics based on A1C despite having normal fasting glucose levels were older. While the International Expert Committee [6] acknowledges that A1C may increase with age based on Pani's work [15], it does not suggest age-specific values in diagnostic scheme. Our results would suggest that this observation deserves further scrutiny. Further investigation and follow-up of individuals with raised A1C in rural and high altitude populations is necessary before adaptation of the new recommendations in Peru.

Our observations regarding the agreement between the two criteria are similar to a recent study from US adult population where, overall , A1C≥6.5% showed fair agreement (κ 0.40) with fasting glucose for diagnosing diabetes [16]. Such agreement values would mean that the test is only moderately good for positive diagnosis or ruling-in disease [14], [17].

Over half of the subjects classified as diabetes cases using A1C, would be considered normal using fasting glucose. The group with elevated A1C but normal fasting glucose was older and socioeconomically deprived, however did not exhibit any of the other classic risk factors for metabolic and cardiovascular disease other than higher blood pressure levels. Using A1C also classified more people living in rural areas as diabetes cases; these results may indicate true disease prevalence in rural areas, reflect genetic differences, or the effects of altitude on A1C. Indeed, a recent genome-wide association study by Soranzo et al. showed that most gene variants that affect A1C levels are likely to do so via erythrocyte biology rather than glycaemic pathways [18]. As for altitude, one of its known effects is hyperemia, yet no differences in hemoglobin levels and anemia were observed in the groups of interest.

Further studies are needed to confirm our findings given their major implications in low income settings, where rural areas will struggle to manage chronic conditions with limited resources. A1C is not limitations-free and, at the individual level, these are related to hemoglobin traits, red cell turnover, age and racial disparities. Further limitations with the test itself do exist, particularly related to their high cost and need of standardization [6]. In the present study, despite the relatively small number of diabetes cases, we were able to detect differences between the groups, that is, the study was not underpowered to evaluate the differences under scrutiny.

Our interpretations may be limited by the cross-sectional nature of the study; we cannot infer the differences or relationships observed are causal without appropriate longitudinal data in our population. The study could have been strengthened with oral glucose tolerance test (OGTT) results. The DECODE Study Group has reported that, compared to fasting glucose, the use of this gold standard would yield a true prevalence 30 to 60% higher [19], [20], [21]. However, even our estimates obtained using fasting glucose had been 30–60% higher through use of an OGTT, this increased prevalence would still be much lower than the prevalence of diabetes we identified through using A1C. It is unlikely that these results are due to inadequate fasting, if this was the case, we would have observed cases where fasting glucose was elevated but A1C was normal.

Our findings may have major implications for determining the burden of diabetes in LMIC. The increased prevalence of diabetes using the A1C cut-offs, could potentially increase health care costs and may place patients at risk of unnecessary drug-related side-effects. While there is evidence from the United States that elevated A1C is linked to CVD mobility [7], further studies are needed to determine whether elevated A1C is related to increased diabetic complications and/or the development of cardiovascular disease, before A1C is recommended as a diagnostic criterion for LMIC. In addition, it needs to be determined whether it is appropriate to intervene on A1C in these settings, independently of glucose levels.

### Conclusions

In conclusion using A1C to define diabetes tripled its prevalence; the increase being more marked among poorer and rural populations. This study suggests the use of A1C as diagnostic criteria for diabetes may have major implications for the burden of disease in LMIC.
